# Comparison of lipases and glycoside hydrolases as catalysts in synthesis reactions

**DOI:** 10.1007/s00253-016-8055-x

**Published:** 2016-12-19

**Authors:** Patrick Adlercreutz

**Affiliations:** Department of Chemistry, Division of Biotechnology, Lund University, P.O. Box 124, 221 00 Lund, Sweden

**Keywords:** Lipase, Glycoside hydrolase, Transesterification, Transglycosylation

## Abstract

Lipases and glycoside hydrolases have large similarities concerning reaction mechanisms. Acyl-enzyme intermediates are formed during lipase-catalyzed reactions and in an analogous way, retaining glycoside hydrolases form glycosyl-enzyme intermediates during catalysis. In both cases, the covalent enzyme intermediates can react with water or other nucleophiles containing hydroxyl groups. Simple alcohols are accepted as nucleophiles by both types of enzymes. Lipases are used very successfully in synthesis applications due to their efficiency in catalyzing reversed hydrolysis and transesterification reactions. On the other hand, synthesis applications of glycoside hydrolases are much less developed. Here, important similarities and differences between the enzyme groups are reviewed and approaches to reach high synthesis yields are discussed. Useful strategies include the use of low-water media, high nucleophile concentrations, as well as protein engineering to modify the selectivity of the enzymes. The transglycosylases, hydrolases which naturally catalyze mainly transfer reactions, are of special interest and might be useful guides for engineering of other hydrolases.

## Introduction

Specialty lipids and carbohydrates are important targets for synthesis reactions. Their synthesis in living cells is normally catalyzed by transferases in reactions involving activated substrates. For example, glycosyl transferases use nucleotide-activated sugars to synthesize oligosaccharides and acyl transferases use acyl-coenzyme A in building up triacylglycerols. These enzymes have high specificity, and due to the high energy of the activated substrates, the reactions are virtually irreversible. However, for in vitro synthesis applications in laboratories and industry, the use of activated substrates is usually not practical because of high costs. Furthermore, many of the transferases are not suitable for applications due to difficulties in production and poor stability. On the other hand, the enzymes that degrade oligosaccharides and lipids in vivo, the hydrolases, are robust enzymes, often well suited for practical applications. The most straightforward way to use hydrolases for synthesis is to carry out *reversed hydrolysis* (condensation) reactions, but this requires that the water concentration is substantially reduced compared to most aqueous solutions with water concentrations of about 55 M, and therefore, it is important to find enzymes which are active under those conditions. There are many reports on enzymes working well in predominantly organic media, as long as some water remains bound to the enzyme (Carrea and Riva 2008; Gupta 1992; Klibanov 2001). Another possibility, which does not necessarily require low water content, is to use hydrolases in kinetically controlled *transfer reactions*. Many hydrolases work according to mechanisms involving a covalent enzyme-substrate intermediate, such as an acyl-enzyme, and have the potential to carry out transfer reactions in addition to the normal hydrolysis.

Lipases have been used very successfully for many synthesis applications (Adlercreutz 2013), but it is considerably more difficult to make glycoside hydrolases work well for synthesis, despite the similarities between the two enzyme types. This review will discuss similarities and differences between the enzyme types and give guidelines on how to improve the possibilities to use more hydrolases for synthesis applications.

## Reaction mechanisms

**Fig. 1:**
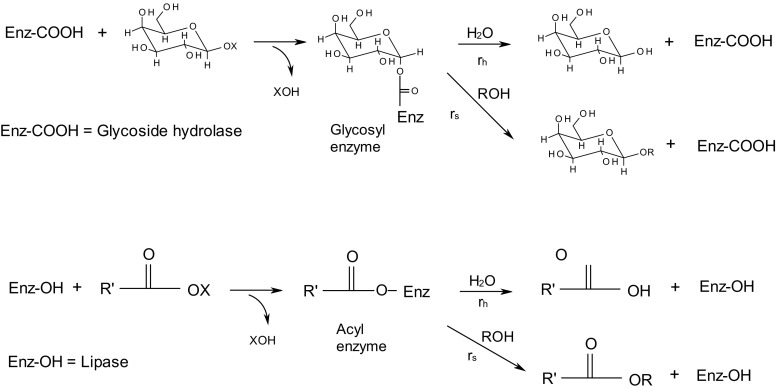
Reaction mechanisms. Glycosylation of a glycoside hydrolase forms a glycosyl-enzyme intermediate and a acylation of a lipase forms an acyl-enzyme intermediate. These intermediates can react with water in hydrolysis reactions (rate: *r*_h_) or with an alcohol in transfer reactions (rate: *r*_s_)

The active site of typical lipases contains a catalytic triad involving a serine residue, which takes part in the formation of an acyl-enzyme intermediate during the reaction (Schmid and Verger 1998) (Fig. 1). The acyl-enzyme can not only react with water causing the normal hydrolysis reaction but also with other nucleophiles, leading to transesterification reactions and the formation of ester products. In the catalytic mechanism of retaining glycoside hydrolases, a covalent glycosyl-enzyme intermediate is formed (Zechel and Withers 2000). This can not only react with water but also with other nucleophiles, giving glycosides as products (Fig. 1). The acyl donor in transesterification reactions is an ester, and in the transglycosylation reactions, the donor substrate is a glycoside, typically a disaccharide or a nitrophenyl glycoside. Reversed hydrolysis reactions can give the same products as the transfer reactions, but in this case, the acyl-enzyme intermediate is formed from a free carboxylic acid and the lipase, while the glycosyl-enzyme intermediate is formed from a carbohydrate, typically a monosaccharide, and the glycoside hydrolase (Fig. 1). It is worth pointing out that alcohols can act as nucleophiles both for lipases and for glycoside hydrolases. To facilitate comparisons of the two enzyme types, alcohol nucleophiles will be used as the main example in this review. The products of these reactions are esters and alkyl glycosides, which are products of considerable practical interest.

## Reversed hydrolysis and transfer reactions

Reversed hydrolysis reactions are under thermodynamic control, and the equilibrium constant is thus of prime importance:

1 $$ {K}_{\mathrm{eq}}=\frac{\left[\mathrm{Product}\right]\left[\ {\mathrm{H}}_2\mathrm{O}\right]}{\left[\mathrm{Substrate}\right]\left[\mathrm{Alcohol}\right]} $$

If a high ratio between the product and the substrate is desired, it is thus beneficial to use a high alcohol concentration and a low water concentration:

2 $$ \frac{\left[\mathrm{Product}\right]}{\left[\mathrm{Substrate}\right]}={K}_{\mathrm{eq}}\times \frac{\left[\mathrm{Alcohol}\right]}{\left[{\mathrm{H}}_2\mathrm{O}\right]} $$

On the other hand, transfer reactions, like transesterification and transglycosylation, can be carried out under kinetic control, thus making it possible to reach product concentrations higher than those present at equilibrium, provided the kinetics are favorable. There are different ways to quantify the ability of the enzyme to catalyze transfer reactions. It is often useful to relate the rate of the transfer (synthesis) reaction (*r*_s_) to the rate of the hydrolysis reaction (*r*_h_). Most simply, this is done by just stating the ratio between the two reactions: *r*_s_/*r*_h_. The *r*_s_/*r*_h_ ratio is strongly influenced by the concentrations of the potential nucleophiles. Selectivity factors (*S*_c_) take this into consideration and thus constitute a better way of characterizing the enzyme than just stating *r*_s_/*r*_h_ (Hansson et al. 2001). Instead of using concentrations, thermodynamic activities can be used to quantify the amounts of the nucleophiles (Halling 1994), and selectivity factors based on activities (*S*) can be defined as well (Van Rantwijk et al. 1999):

3 $$ \frac{r_{\mathrm{s}}}{r_{\mathrm{h}}}={S}_{\mathrm{c}}\times \frac{\left[\mathrm{Alcohol}\right]}{\left[{\mathrm{H}}_2\mathrm{O}\right]} $$

4 $$ \frac{r_{\mathrm{s}}}{r_{\mathrm{h}}}=S\times \frac{a_{\mathrm{alcohol}}}{a_{\mathrm{w}}} $$

It should be pointed out that the selectivity constants are influenced by reaction conditions in general, for example, the water activity. The water activity thus has a double effect: both as reactant in hydrolysis and as modulator of enzyme properties and thereby the selectivity constants. Still, another way to quantify the ability of hydrolases to catalyze transfer reactions was recently proposed for glycoside hydrolases working in predominantly aqueous solutions. The new descriptor is defined as the nucleophile concentration giving equal rates of transglycosylation and hydrolysis and is called T_50_ (Mangas-Sanchez and Adlercreutz 2015).

A general way to favor synthesis reactions with alcohols by hydrolases is to use low water concentration and/or high alcohol concentration, which will be reviewed in the coming two sections. Thereafter, selectivity of different types of hydrolases for transfer and hydrolysis reactions will be compared and protein engineering efforts to improve the selectivity of glycoside hydrolases for transfer reactions will be reviewed.

## Use of low-water media to achieve high synthesis yields

**Fig. 2:**
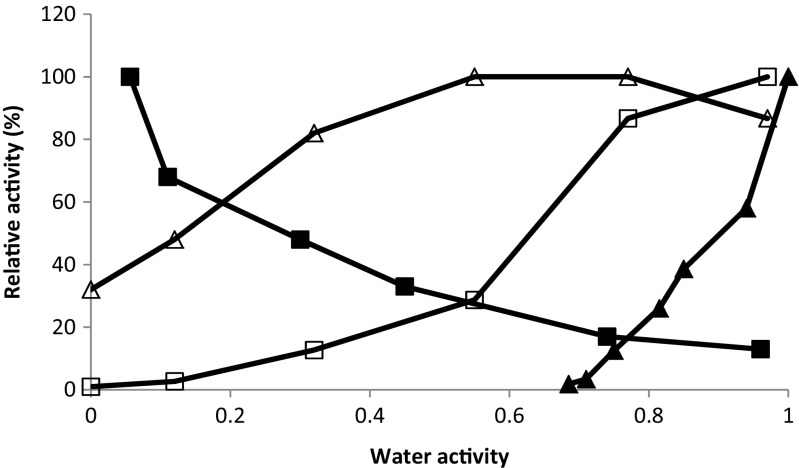
Water activity dependence. The catalytic activity of various enzymes is shown as a function of the water activity: *Candida antarctica* lipase B (■; Nordblad and Adlercreutz 2013), *Rhizomucor miehei* lipase (△; Valivety et al. 1992b), *Burkholderia cepacia* lipase (□; Valivety et al. 1992b), and almond β-glucosidase (▲; Ljunger et al. 1994)

Among the lipases, there is large variation with respect to water activity dependence (Valivety et al. 1992b) (Fig. 2). Some lipases, such as the *Burkholderia cepacia* lipase (previously called *Pseudomonas cepacia* lipase), behave like most other enzymes expressing increasing catalytic activity with increasing water activity (Wehtje and Adlercreutz 1997). On the contrary, *Rhizopus* lipases and lipase B from *Candida antarctica* express maximal catalytic activity at low water activity (Nordblad and Adlercreutz 2013; Wehtje and Adlercreutz 1997), and *Rhizomucor miehei* lipase has been shown to be catalytically active even at a water activity as low as 0.0001 (Valivety et al. 1992a). For some lipases, there are thus very good possibilities to achieve efficient synthesis reactions by using low-water media (Ma et al. 2002).

The number of glycoside hydrolases studied at low water activity is limited, but the reports indicate increasing activity with increasing water activity and relatively high water activity is needed to observe any catalytic activity: 0.67 for almond β-glucosidase (Ljunger et al. 1994) (Fig. 2) and 0.29 for *Pyrococcus furiosus* β-glycosidase (Hansson and Adlercreutz 2001).

The reasons for the large differences in water activity dependence among the enzymes are not known, but it is likely that some water molecules bound to the enzyme have crucial functions and if these molecules are removed, the enzyme loses its activity. It has been shown that glycoside hydrolases belonging to family 1 have structures including highly conserved water molecules (Teze et al. 2013). Seven water molecules were found in more than 90% of the known structures, and several more water molecules are present in more than 60% of the structures. These conserved water molecules are often arranged in clusters, some of which seem to form water channels, which can aid in transporting water molecules from the bulk solution to the active site.

Engineering of glycoside hydrolases to become active at low water activity is of interest, for example, for the synthesis of alkyl glycosides. It is likely that multiple changes are needed to make an enzyme active at low water activity; a single mutation will probably not give the desired effect. Considering the limited information on the function of water molecules associated with enzymes, it seems to be a very challenging task to engineer them for low water activity. On the other hand, some progress has been made by immobilization of glycoside hydrolases on suitable supports. When two *Thermotoga neapolitana* β-glucosidases were immobilized by adsorption on a porous polypropylene support, they expressed 17–70 times higher activity at low water activity (0.7) than freeze-dried enzyme, but had comparable or lower activity at full hydration (Lundemo et al. 2014). A possible explanation could be that immobilization promoted the retention of crucial water molecules bound to the enzyme, thereby helping it to retain catalytic activity at low water activity. A similar mechanism has been proposed for the increased activity of the protease α-chymotrypsin at low water activity by addition of polyols and saccharides (Adlercreutz 1993).

## Use of high nucleophile (alcohol) concentrations to achieve high yields

Most lipases are strongly inhibited by high alcohol concentrations (Janssen et al. 1999; Ma et al. 2002), which means that the enzyme is used quite inefficiently. High lipase loadings can make it possible to achieve high yields anyway, and it should be pointed out that esters can be successfully produced by lipase-catalyzed esterification in solvent-free systems (Petersson et al. 2005). Alternatively, low alcohol concentration can be used for transesterification reactions catalyzed by lipases having high enough selectivity for the alcohol (see the following sections).

On the other hand, high alcohol concentration is advantageous for glycoside hydrolase-catalyzed synthesis of alkyl glycosides both by reversed hydrolysis and by transglycosylation. Several glycoside hydrolases have been used successfully in reaction media primarily consisting of an alcohol, such as hexanol or octanol. These alcohols are not miscible with water, so there is normally an aqueous phase containing the enzyme and hydrophilic substrates (carbohydrates, etc.). The aqueous phase is thus saturated with the alcohol, which is beneficial for synthesis. This type of reaction works fairly well with alcohols with a chain length up to about eight carbon atoms. For longer alcohols, slow reactions and poor yields are obtained probably because of poor solubility of the alcohol in the aqueous phase.

In conclusion, high alcohol concentration is beneficial for glycoside hydrolase-catalyzed reactions but not for lipase-catalyzed ones. Probably, the alcohol competes with binding of the acyl donor, thereby interfering with the acylation step of lipase catalysis (Janssen et al. 1999).

## Transfer/hydrolysis selectivity of lipases and glycoside hydrolases

The acyl-enzyme and glycosyl-enzyme intermediates (Fig. 1) can react with water (hydrolysis) or with other nucleophiles (transfer reaction). The competition between the transfer reaction and the hydrolysis is influenced to a large extent by the properties of the enzyme. The selectivity can be quantified in terms of selectivity factors (*S*, defined in Eq. 4). Table 1 shows *S* values from published studies of various lipases and glycoside hydrolases. To make a comparison between lipases and glycoside hydrolases, examples involving 1-hexanol, or in a few cases other alcohols, have been selected. The *S* values observed in reactions catalyzed by glycoside hydrolases vary considerably, but they are all much lower than those reported for lipases. It would thus be highly beneficial for synthesis applications if glycoside hydrolases could be modified to express selectivity similar to those of lipases.

**Table 1:** Selectivity factors (S, defined in Eq. 4) reported from reactions catalyzed by various lipases and glycoside hydrolases involving 1-hexanol or other alcohols as acceptor substrates

Enzyme	Donor substrate	Acceptor	S	Reference
*Rhizopus oryzae* lipase	Ethyl decanoate	1-Hexanol	350	Ma et al. (2002)
*Candida rugosa* lipase	Ethyl decanoate	1-Hexanol	230	Ma et al. (2002)
*Candida antarctica* lipase B	Ethyl acrylate	1-Octanol	43	Nordblad and Adlercreutz (2013)
*Pyrococcus furiosus* β-glycosidase	Pentyl-β-glucopyranoside	1-Hexanol	2	Hansson and Adlercreutz (2001)
*Sulfolobus solfataricus* β-galactosidase	*p*-nitrophenyl-β-glucopyranoside	1-Hexanol	2.6	Hansson et al. (2001)
Almond β-glucosidase	*p*-nitrophenyl-β-glucopyranoside	1-Hexanol	0.7	Hansson et al. (2001)
*Escherichia coli* β-galactosidase	*p*-nitrophenyl-β-galactopyranoside	1-Hexanol	9	Hansson et al. (2001)
*S. solfataricus* β-galactosidase	*p*-nitrophenyl-β-galactopyranoside	1-Hexanol	6	Hansson et al. (2001)
Almond β-glucosidase	*p*-nitrophenyl-β-galactopyranoside	1-Hexanol	8	Hansson et al. (2001)
*Thermotoga neapolitana* β-glucosidase 1A	*p*-nitrophenyl-β-glucopyranoside	1-Hexanol	0.5	Lundemo et al. (2016)
Invertase	Sucrose	1-Butanol	0.6	Van Rantwijk et al. (1999)
β-Xylosidase	Methyl-β-xyloside	1-Propanol	7	Van Rantwijk et al. (1999)

## Engineering glycoside hydrolases

Directed evolution has been shown to be a useful strategy for obtaining glycoside hydrolases with improved selectivity for transglycosylation compared to hydrolysis. A β-glycosidase from *Thermus thermophilus* modified in this way produced 60 and 75% transglycosylation yield from maltose and cellobiose, respectively, compared to 6 and 8% for the wild-type enzyme (Feng et al. 2005). X-ray structures of wild-type and mutants with improved selectivity for transglycosylation vs. hydrolysis were compared, and no major structural changes were observed (Teze et al. 2014). It was noted that several mutations having a positive effect on the observed transglycosylation/hydrolysis ratio occurred in highly conserved residues in the −1 binding site (Teze et al. 2014). Mutating such residues, in *T. thermophilus* β-glycosidase, produced four new mutants with increased selectivity for transglycosylation. Mutation of conserved residues in the −1 site might thus be a general, semi-rational strategy for improving the *r*_s_/*r*_h_ ratio of reactions catalyzed by this group of enzymes (Teze et al. 2014).

The transglycosylation/hydrolysis ratio in reactions involving hexanol as nucleophile catalyzed by β-glucosidase from *P. furiosus* was increased by more than a factor of two by a single mutation (F426Y) (Table 2) (Hansson and Adlercreutz 2001). The corresponding ratio for *T. neapolitana* β-glucosidase 1A was increased by a factor of about seven by a single mutation (N220F), thereby increasing the expected hexyl glucoside yield from 17 to 58% (Lundemo et al. 2013). Further improvements were achieved by modulation of pH during the reaction. The pH-activity curve for hydrolysis was bell shaped both for the wild-type and the mutant enzyme, and the apparent p*K*a values of the catalytically active amino acids (the nucleophile and the acid-base) did not differ significantly between the two enzyme variants. However, increasing the pH to ten hardly affected the transglycosylation activity of the N220F mutant (and two other mutants) while their hydrolysis activity was largely eliminated, thus making it possible to carry out transglycosylation with good rate without interfering hydrolysis (Lundemo et al. 2016).

**Table 2:** Approaches to reach high synthesis yields

Approach	Lipases	Glycoside hydrolases
Reversed hydrolysis		
Low water concentration	+++	+
High alcohol concentration	++	++
Enzyme selectivity	–	–
Transfer reaction		
Low water concentration	+++	+
High alcohol concentration	+	++
Enzyme selectivity	+++	+ 1) +++2)

The degree of success of different approaches is estimated using the following scale: +++, excellent results obtained; ++, good results obtained; +, moderate success so far; –, this approach does not work; 1), can be improved for some glycoside hydrolases; 2) transglycosylases

Removal of the nucleophile of the active site of glycoside hydrolases has been used to create the so-called glycosynthases, which can be used for synthesis of glycosidic bonds but are unable to catalyze hydrolysis. To make the reaction occur, an activated donor substrate, typically a fluoride, is often used. The glycosidic bond is formed in a single displacement mechanism. Alternatively, an external nucleophile, typically formate, is used together with a normal donor substrate in a double displacement reaction. Since glycosynthases do not catalyze hydrolysis, the glycosidic products formed are not degraded, which is a great advantage. Glycosynthases will not be treated in detail here; for a good review, see Perugino et al. (2004).

## Natural transglycosylases

Some enzymes have strong homology with normal glycoside hydrolases but catalyze mainly transglycosylation reactions (Bissaro et al. 2015). One example is the glucansucrases, classified in glycoside hydrolase family 70. These enzymes catalyze the transfer of d-glucopyranosyl residues from sucrose to an acceptor, resulting in polysaccharides like dextran, reuteran, and alternan (Monsan et al. 2010). The enzymes are quite promiscuous with respect to acceptors; various monosaccharides and carbohydrates have been found to be accepted.

Glycoside hydrolase family 13 includes enzymes with quite variable ability to catalyze transglycosylation. The α-amylases constitute a large subgroup in this family, and they catalyze mainly hydrolysis. Cyclodextrin glucanotransferases (CGTases) within this family are closely related with the α-amylases, but many of them catalyze mainly intramolecular transglycosylation reactions producing cyclodextrins from starch. Compared to the α-amylases, the CGTases have extra positive subsites, which can bind acceptor substrates, including the nonreducing end of the oligosaccharide being cleaved off the starch molecule. Mutations in these subsites can cause a severe reduction in transglycosylation activity (van der Veen et al. 2001). Interestingly, just three mutations converted a CGTase to an α-amylase-like enzyme with high hydrolysis activity and virtually no ability to produce cyclodextrins (Kelly et al. 2007).

Xyloglucans are polysaccharides with important functions in plant cell walls. Interestingly, glycoside hydrolase family 16 includes enzymes, which are specialized in catalyzing either hydrolysis or transglycosylation of xyloglucans (Eklof and Brumer 2010). The transglycosylation reactions are of physiological importance in the remodelling of the cell walls in the development of the plants. Detailed structural analysis has revealed differences concerning loops lining the substrate-binding cleft, but largely, the structures of these two types of enzymes are surprisingly similar. It is thus clear that subtle differences in structure can induce dramatic changes in the ratio between transglycosylation and hydrolysis activities.

## Concluding remarks

Approaches for achieving high synthesis yields in synthesis reactions catalyzed by lipases and glycoside hydrolases are summarized in Table 2. A major advantage of lipases is their good activity at low water activity which is favorable both for reversed hydrolysis and transesterification. Increasing the alcohol concentration causes inhibition of lipases, but of course, the effect on the equilibrium is positive which is especially important in reversed hydrolysis. Furthermore, many lipases have excellent selectivity for reactions with other nucleophiles than water, with alcohols as good examples.

Synthesis applications of glycoside hydrolases are hampered by their low catalytic activity at low water activity. High alcohol concentrations can be beneficial and make it possible to achieve reasonable yields with alcohols up to about eight carbon chain length. The selectivity of glycoside hydrolases varies substantially. For many enzymes of this type, selectivity is rather poor and decreases further if the water activity is reduced. Protein engineering has proven to be very useful for improving transglycosylation activities. Great progress has been made in the conversion of glycosidases to glycosynthases. Furthermore, the existence of natural transglycosidases is inspiring. These enzymes are very closely related to normal glycoside hydrolases but catalyze almost exclusively transglycosylation. If the reasons behind these activity changes could be fully understood, it would improve the possibilities to use glycoside hydrolases for synthesis dramatically.
